# Phase 1 Trial of Autologous Bone Marrow Stem Cell Transplantation in Patients with Spinal Cord Injury

**DOI:** 10.1155/2016/6768274

**Published:** 2016-07-05

**Authors:** Zurab Kakabadze, Nickolas Kipshidze, Konstantine Mardaleishvili, Gocha Chutkerashvili, Irakli Chelishvili, Albrecht Harders, George Loladze, Gocha Shatirishvili, Nodar Kipshidze, David Chakhunashvili, Konstantine Chutkerashvili

**Affiliations:** ^1^Department of Clinical Anatomy, Tbilisi State Medical University, 0177 Tbilisi, Georgia; ^2^N. Kipshidze Central University Hospital, 0177 Tbilisi, Georgia; ^3^Department of Endovascular Therapy, New York Cardiovascular Research, New York, NY 10128, USA; ^4^Department of Cell Therapy and Cancer Immunotherapy, Cancer Research Centre, 0177 Tbilisi, Georgia; ^5^Department of Neurosurgery, Archangel St. Michael Multi Profile Clinical Hospital, 0112 Tbilisi, Georgia; ^6^Department of Neurosurgery, Ruhr University Bochum, 44801 Bochum, Germany; ^7^College of Global Public Health, New York University, New York, NY 10003, USA

## Abstract

*Introduction.* A total of 18 patients, with complete motor deficits and paraplegia caused by thoracic and lumbar spine trauma without muscle atrophy or psychiatric problems, were included into this study.* Materials and Methods.* The bone marrow was aspirated from the anterior iliac crest under local anesthesia and the mononuclear fraction was isolated by density gradient method. At least 750 million mononuclear-enriched cells, suspended in 2 mL of saline, were infused intrathecally.* Results and Discussion.* The study reports demonstrated improvement of motor and sensory functions of various degrees observed in 9 of the 18 (50%) cases after bone marrow stem cell transplantation. Measured by the American Spinal Injury Association (ASIA) scale, 7 (78%) out of the 9 patients observed an improvement by one grade, while two cases (22%) saw an improvement by two grades. However, there were no cases in which the condition was improved by three grades.* Conclusions.* Analysis of subsequent treatment results indicated that the transplantation of mononuclear-enriched autologous BMSCs is a feasible and safe technique. However, successful application of the BMSCs in the clinical practice is associated with the necessity of executing more detailed examinations to evaluate the effect of BMSCs on the patients with spinal cord injury.

## 1. Introduction

Spinal cord injury is a disorder that often causes severe disability, such as incomplete or complete tetraplegia or paraplegia. The economic burden relative to the estimated lifetime costs for treatment and healthcare of one patient could be as high as 4.5 million USD. According to the National Spinal Cord Injury Statistical Center, the annual incidence of spinal cord injury in the USA is 40 cases per million, including those who have survived the accident. Annually the crude incidence is 12,500 new cases [1]. There is neither a cure for the disorder nor any effective treatment for patients with injured spinal cords. The main surgical procedure is a decompression of the spinal cord in addition to a high dose of methylprednisolone [2].

Though early decompression could have a neuroprotective effect, less than 1% of patients showed complete neurological recovery at hospital discharge. Many patients remain in a wheelchair. Pharmacological agents such as methylprednisolone [3, 4], naloxone, monosialotetrahexosylganglioside (GM-1), or TRH were studied in clinical trials with no treatment demonstrating strong evidence for clinical benefits [5]. Autologous stem cells could help the regeneration of an injured spinal cord. Bone marrow mesenchymal and hematopoietic stem cells have differentiation potential. There are findings that BMSCs differentiate into mature neurons or glial cells under experimental conditions [6, 7]. It was demonstrated that mesenchymal stem cells could differentiate into neuronal-like cells* in vivo*, which express the neural cell marker. Preclinical studies have shown that such differentiated cells were able to improve or restore damaged spinal cord function. Replacement, dedifferentiation, or paracrine effects were suggested. These findings demonstrated that the use of BMSCs has a therapeutic potential in patients with neurological diseases.

Different cell types were used in preclinical studies for SCI treatment. NSCs, MSCs, ESCs, OECs, SCs, and iPSCs have all proved that they have regenerative potential [8]. MSCs, specifically, have low immunogenicity and possess anti-inflammatory and immunosuppresive effects [9]. Bone marrow-derived MSCs are the most widely used stem cells for SCI experiments. They differentiate into neurons and glia cells [10]. Some authors suggest that cell fusion and transdifferentiation are the main mechanisms [11–13]. In animal models BM MSCs were injected into spinal cord injury area [14] or intrathecally with some effects [15]. Experiments in nonhuman primates and pigs demonstrated successful mean improvement of locomotor function [16, 17]. The secretion of growth factors and anti-inflammatory cytokines has been proposed as the main mechanism in cell transplantation [18–20]. Clinical trials showed safety and feasibility of BM MSCs transplantation for SCI patients. No serious complications were reported and patients showed improvement of motor and sensory functions [21–23]. The objective of this study was to assess the safety and efficacy of transplanting of autologous bone marrow stem cell in patients with spinal cord injury.

## 2. Materials and Methods

### 2.1. Patients

Starting from March of 2012 until December of 2014, a total of 18 patients, with complete motor deficits and paraplegia caused by thoracic and lumbar spine trauma without muscle atrophy or psychiatric problems, were included into this study. All patients signed a written informed consent. The study protocol was confirmed according to ethical guidelines of the 1975 Declaration of Helsinki and was approved by Archangel St. Michael Multi Profile Clinical Hospital, Tbilisi, Georgia. After Ethics Committee approval, patients between the ages of 18 and 65 years and of either gender were preliminary candidates. Inclusion criteria of subjects were as follows: subjects have chronic spinal cord injury (>6 months after initial spinal cord injury surgery) who have stable neurological symptoms for at least 6 months; subjects have current neurological status of ASIA score A; the location of neurological injury of the patient is between C5 and T11; the injured site of the spinal cord is within three vertebral levels; subjects must be able to read, understand, and complete the VAS; and subjects have voluntarily signed and dated an informed consent form prior to any study procedures. Those screened were excluded on the basis of the following criteria: anatomical transection of the spinal cord; spinal cord lesion by sharp objects; ongoing infections; terminal, neurodegenerative, or primary hematological diseases; osteopathy which might increase the risk of spinal cord puncture; coagulopathies; severe hepatic, renal, or heart failure; and pregnancy or lactation.

### 2.2. Bone Marrow Cell Therapy

100–120 mL of bone marrow was aspirated from the anterior iliac crest under local anesthesia and placed in sterile tubes containing heparin. The aspirates were diluted 1 : 2 with PBS. The mononuclear fraction was isolated by density gradient centrifugation at 400 ×g for 30 min at room temperature using Ficoll Paque Plus or Ficoll Paque Premium solution (GE Healthcare, USA). At least 750 million mononuclear-enriched cells, suspended in 2 mL of saline, were infused intrathecally.

### 2.3. Flow Cytometry and Viability Testing

0.4 mL of the final cell product was subjected to trypan blue dye exclusion test and flow cytometric analysis. Viability test was performed by 0.4% trypan blue solution (Sigma, USA) according to standard protocol. For cell immunophenotyping cell suspensions were incubated with anti-human CD34, anti-human CD45, and antihuman CD 271 (all from Miltenyi Biotec, Germany) and anti-human-STRO-1 antibodies (Santa-Cruz Biotechnology, USA) in 0.5% BSA/PBS (Sigma, USA) buffer in accordance with manufacturer's instructions. Flow cytometry analysis was carried out on BD FACSCalibur flow cytometer (Becton Dickinson, USA).

**Figure 1:**
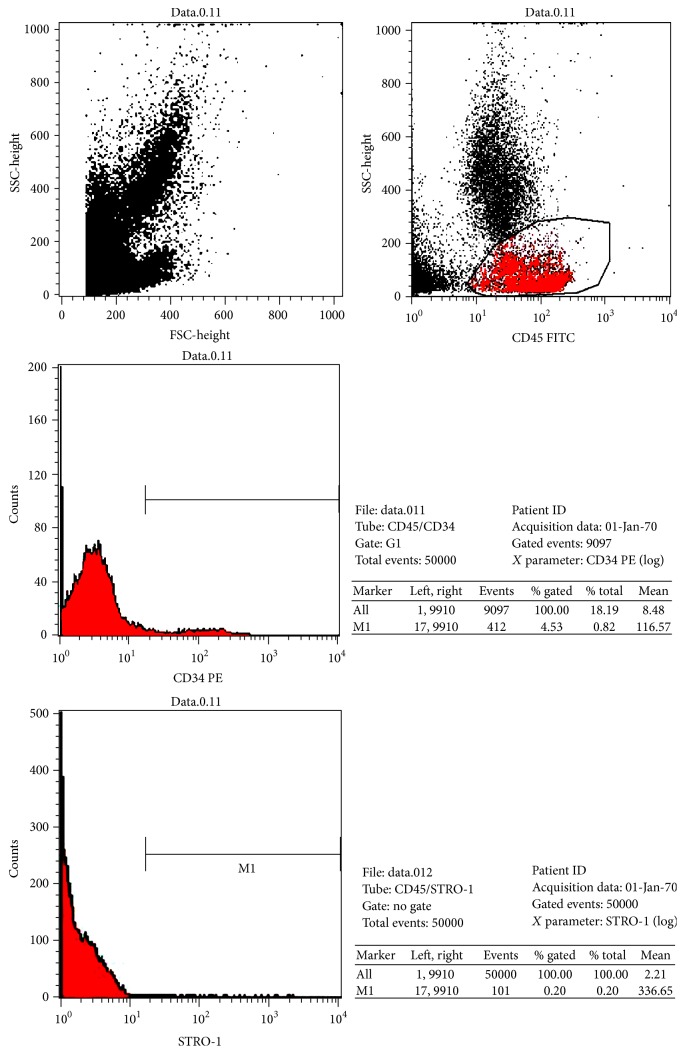
Representative picture of flow cytometric analysis. Mononuclear CD45−/CD34−/CD271+/STRO-1+ cells defined as bone marrow mesenchymal stem cell and their enumerated percentage and absolute count. Bone marrow hematopoietic stem cells determined in CD45+/CD34+ mononuclear cell population and their enumerated percentage and absolute count.

Mononuclear CD45−/CD34−/CD271+/STRO-1+ cells were defined as BM MSCs and their percentage and absolute count were enumerated. Bone marrow hematopoietic stem cells were determined in CD45+/CD34+ mononuclear cell population and their percentage and absolute counts were enumerated (Figure 1). The total amount of autologous bone marrow and the detailed number of final bone marrow stem cell products are shown in Table 1.

### 2.4. Follow-Up Period

The follow-up visits were scheduled for 12 months after transplantation. During every follow-up visit, preoperatively, and after 3 and 6 months after surgery, the results were evaluated by assessing ASIA impairment scale, measuring electrophysiological parameters, including electroneuromyography and enhanced MRI.

### 2.5. Statistical Analysis

Statistical analysis was performed by using SPSS Statistics v20 software. A paired sample *t*-test was used for determining whether or not there is a statistically significant difference between the results acquired before and after the treatment. Initial data (before treatment) was compared to that acquired at 6 and 12 months after treatment was performed. A significance level of 0.05 was chosen.

## 3. Results and Discussion

Transplantation of bone marrow stem cells was performed on 18 patients, among which 13 were male (72%) and 5 were female patients (28%), aged 22 to 65. Patients' profiles are shown in Table 2. There were 12 (67%) patients with injury of the thoracic spine and 6 (33%) patients with lumbar spine. The period of time that has passed since the SCI was from 5 to 20 months. According to the ASIA classification of SCI, there were 10 cases (56%) with ASIA A score, 7 of which were males and 3 were females; five cases (28%) had B score, 4 of these were males and 1 was female; and there were 3 (17%) cases with ASIA C score, out of which 2 were males and 1 was female.

Out of the 18 total patients, the reasons of SCI in 11 (61%) of the cases were car accidents, 4 (22%) cases due to falling and 3 (17%) cases due to a gunshot wound. All procedures were performed without any specific side effects or complications except for mild pain in the anterior iliac crest region at the sites of bone marrow puncture. Headaches were observed in 9% of the patients and temperature increased up to 37.5°C in 6% of patients, which lasted for two days. No other complications or specific side effects related to the infusion procedure were reported.

Continuous patient monitoring was carried out during the first 24 hours after transplantation. Following clinical observation patients were discharged. In this study, our attention was mainly focused on assessing the safety of this method. The ASIA score and nerve conduction study reports demonstrated improvement of motor and sensory functions of various degrees observed in 9 of the 18 cases (50%) after bone marrow stem cell transplantation. Measured by the ASIA scale, 7 (78%) out of the 9 patients observed an improvement by one grade. While two cases (22%) saw an improvement by two grades. However, there were no cases in which the condition was improved by three grades. Of the 18 total patients with SCI, the significant damage of the small and large tibial nerves using ENMG did not occur in 8 (44%) patients, in the form of insertional positive sharp waves and end plate potential, where the bioelectric activity is registered in the lower limb muscles. After cell transplantation, 5 (42%) of the 12 patients suffering from urinary tract dysfunction saw improvements in urinary function of varying degrees. Additionally, 7 (78%) of the 9 patients suffering from intestinal dysfunction demonstrated improved function in various degrees. CT changes were observed in four patients in postoperative months ranging from eight to twelve.

Spinal cord injuries are accompanied by a number of complications, causing death of neurons, degeneration of nerve fibers, hemorrhage, and eventually the absence of complete regeneration in areas of injury. In most of the cases, traditional methods of treatment are very rarely able to restore the lost functions of tissues. However, the use of stem cells in such patients gives hope for the opportunity to achieve functional improvements. Currently, human OPCs, Schwann cells, bone marrow stromal cells, nasal olfactory ensheathing cells, and others are being used for stem cell therapy during spinal cord injuries. Preclinical studies of the human OPCs application have shown that the effects of transplantation included robust white and gray matter sparing at the injury epicenter and, in particular, preservation of motor neurons that correlated with movement recovery [18]. One critical aspect of successful cell-based SCI therapy is the time of injection following injury [19]. The authors note that they injected the majority of transplanted OPCs at two clinically relevant times when most damage occurs to the surrounding tissues, 3 and 24 hours following injury.

The derived OPCs expressed oligodendrocyte markers, including 2′,3′-cyclicnucleotide 3′-phosphodiesterase, galactocerebroside, oligodendrocyte transcription factor (Olig1), and oligodendrocyte markers (O4 and O1). Moreover, OPCs survived when injected at the center of injury and migrated away from the injection sites after one week. Other authors think that human embryonic stem cell-derived OPCs can be transplanted sooner than conventionally accepted. Transplantation of Schwann cells can also be a promising therapeutic strategy for spinal cord repair. They are one of the most widely studied cell types for repairing the spinal cord. Unlike oligodendrocytes and their precursors, Schwann cells possess many of the characteristics that are desirable for transplantation in spinal cord lesions. They can be easily collected from peripheral nerve and easily purified and grown in culture in large quantities. Due to their ability to dedifferentiate, migrate, proliferate, and express growth promoting factors and myelinate regenerating axons, Schwann cells play a crucial role in endogenous repair of peripheral nerves [20, 21]. The transplantation of Schwann cells in the injured spinal cord boosts the regeneration of axons, myelinates or ensheathes regenerated axons in a normal way, reduces cyst formation in the injured tissue, and reduces secondary damage of the tissues around the initial injury site [22]. In order to improve the clinical condition of the patients with SCI, the cultured and purified autologous Schwann cells, which were previously isolated from the sural nerve, were transplanted [23].

As it is noted by authors, there were some signs of improvement in the autonomic, motor, and sensory function of all patients. Authors report that they have assessed the safety and feasibility of a combination of bone marrow mesenchymal stromal cells and Schwann cells for the treatment of patients with chronic spinal cord injury [24]. However, when transplanting Schwann cells, the regeneration and myelination occur only where the graft is located. The inhibitory nature of the glial scar surrounding the injury axons does not allow for the regeneration of cells beyond the graft [25]. Thus, despite the fact that the Schwann cells having great potential for repairing the injured spinal cord, in order to successfully use them, the researches continue to address issues such as encouraging the survival and growth of damaged axons using neurotrophins, which can help with the establishment of appropriate connections between regenerating axons and target neurons and, thus, provide functional recovery [26], by neutralizing inhibitory molecules associated with the failure of axonal regeneration [27] and many others.

Another promising candidate for cell transplantation in SCI cases may be the olfactory ensheathing cell, considered to be a result of the unique capabilities of these cells as they are being continually replaced throughout one's lifetime, and also the rate of neurogenesis can be regulated by manipulating the system in order to abbreviate or prolong the average life of a sensory neuron [28].

A phase 1, single-blinded clinical trial has shown that, up to one year after implantation, the transplantation of autologous olfactory ensheathing cells into the injured spinal cord is feasible and safe [29]. However, there are contradictory opinions about the effectiveness of transplantation of olfactory ensheathing cells for spinal cord injury. Thus, some authors report that the interfaces of Schwann cells and olfactory ensheathing cells form a 3-dimensional matrix providing a permissive microenvironment for successful axon regeneration in the adult mammalian central nervous system [30]. Others report that the transplantation of Schwann cells or olfactory ensheathing glia, or their combination in the adult Fischer rat thoracic (T9) spinal cord, after 1 week from a moderate contusion, is more effective in promoting axonal sparing/regeneration rather than the combination of Schwann cells with olfactory ensheathing glia or olfactory ensheathing glia graft [31]. Special interest is given to bone marrow hematopoietic and mesenchymal cells. In a phase I/II open-label nonrandomized study, patients were transplanted with BMSCs in acute (within 14 days after injury), subacute (2−8 weeks), and chronic patients (more than 8 weeks). Control group patients were treated with conventional decompression and fusion surgery without BMSC transplantation. At 4 months, the MRI presented spinal cord enlargement without any hemorrhage, new cysts, or infections. The ASIA grade increased up to 30–33% of the acute and subacute treated patients, respectively, (ASIA A to B or C), whereas no significant improvement was observed in the chronic treatment group [32]. In 2007 Sykova and Jendelova published a study on 20 complete spinal cord injury patients. Patients were transplanted 10 to 467 days after injury. Intra-arterial bone marrow cell (via catheterization of a. vertebralis) transplantation was successful in the acute patient group (10–30 days after injury). Patients were evaluated with the ASIA protocol, Frankel score, MRI evaluation, and electrophysiology (MEPs and SEPs). Motor and sensory function were improved in most patients within 3 months. No complications were observed [33]. In 2008 Geffner et al. reported eight cases of treatment of SCI (four acute, four chronic) with bone marrow stem cells. Cells were injected via multiple routes: directly into the spinal cord, intrathecally, and intravenously. For neurological evaluation ASIA, Frankel, and Ashworth scales were used. Comprehensive evaluations demonstrated improvements in ASIA, Barthel (quality of life), Frankel, and Ashworth scoring. ASIA Motor Score/Sensory Light Touch Score/Sensory Pin Prick Score were improved in all 8 patients as well as Barthel Index Score and Bladder Function Score and showed stable improvement even after 2 years after treatment [17].

The similar results were obtained by the authors that transplanted mesenchymal bone marrow stem cells in 40 patients with SCI. The cells were transplanted in the area surrounding the injury. During the whole period of observation there were significant improvements in the patients that had no serious complications [34]. Other authors report that they obtained satisfying results using the transplantation of autologous bone marrow-derived cells in addition to physical therapy in patients with chronic cervical and thoracic SCI; the duration of the injury in these patients was at least 12 months. The injection of stem cells was conducted with intrathecal injection [35]. Others obtained satisfying results when treating the patients with SCI using hematopoietic progenitor stem cells [36]. According to our preliminary research, the prognosis for SCI patients may depend on various reasons, including the etiology of SCI, the time that elapsed since the injury, the age of the patient, the type of stem cells that are more suitable for transplantation in patients with SCI, the amount of cells, the ways to deliver the cells into the lesion, and various other covariates. The further successful application of stem cell therapy in the patients with SCI depends largely on solving the above-mentioned issues.

## 4. Conclusion

Analysis of subsequent treatment results indicated that the transplantation of mononuclear-enriched autologous BMSCs is a feasible and safe technique. Among the adverse effects, patients noted fever and headache, which disappeared within 24–48 hours without intervention. However, successful application of the BMSCs in the clinical practice is associated with the necessity of executing more detailed examinations to evaluate the effect of BMSCs on the patients with spinal cord injury.

## Figures and Tables

**Table 1:** The total amount of autologous bone marrow and the detailed number of final bone marrow stem cell products.

Patients	Bone marrow (mL)	Total cells (×10^6^)	CD45		Mononuclear cells		Mesenchymal cells STRO-1+/CD271+		CD34	
			%	Number of cells (×10^6^)	%	Number of cells (×10^6^)	%	Number of cells (×10^3^)	%	Number of cells (×10^6^)
1	120	536	50	268	28	150	0.04	214	1.5	7.0
2	100	516	53	284	34	176	0.02	103	1.4	7.2
3	100	531	51	271	31	165	0.03	159	1.4	7.4
4	120	542	54	293	38	206	0.04	217	1.5	8.1
5	100	405	55	223	45	181	0.05	713	0.9	3.6
6	100	964	55	530	40	386	0.04	384	1.5	14.5
7	120	620	50	310	37	229	0.03	186	0.8	5.0
8	100	903	58	524	41	375	0.02	181	0.9	8.1
9	100	749	61	457	35	262	0.01	75	1.5	12.0
10	120	902	55	491	29	261	0.02	181	2.5	22.6
11	100	748	61	456	34	261	0.01	74	1.5	11.2
12	100	526	51	267	27	151	0.04	214	1.5	7.9
13	120	512	52	283	33	175	0.02	103	1.4	7.2
14	110	559	81	451	33	187	0.04	210	7.5	41.9
15	100	690	48	330	36	245	0.04	284	2.4	16.6
16	120	542	52	280	27	145	0.05	260	0.9	4.9
17	120	628	49	310	34	214	0.03	210	2.7	17.0
18	100	696	42	294	44	308	0.02	148	2.6	18.1

**Table 2:** Patient demographics.

Patients	Sex	Age	Cause of injury	Injury site	Duration between injury and surgery (months)	ASIA grade
1	M	44	Car accidents	L3-4	20	A
2	M	51	Fall injury	T12–L4	5	A
3	M	24	Gunshot wound	T11-12	10	B
4	F	61	Fall injury	T8	8	A
5	M	38	Car accidents	S3	16	A
6	M	42	Car accidents	T2	7	A
7	M	22	Car accidents	L3-4	12	B
8	M	28	Car accidents	T8	9	B
9	F	54	Fall injury	T12–L3	10	A
10	F	31	Car accidents	L2-3	6	A
11	M	59	Car accidents	T10–12	14	C
12	M	44	Car accidents	L3	19	C
13	M	22	Gunshot wound	L2-3	11	A
14	F	31	Car accidents	T4	10	B
15	F	46	Car accidents	T5-6	17	C
16	M	20	Car accidents	T10–12	20	B
17	M	32	Fall injury	T8	14	A
18	M	19	Gunshot wound	T11-12	10	A

## Abbreviations

ASIA: American Spinal Injury Association
BM MSC: Bone marrow mesenchymal stem cell
BMSC: Bone marrow stem cell
ENMG: Electroneuromyography
ESC: Embryonic stem cell
GM-CSF: Granulocyte-macrophage colony-stimulating factor
iPSC: Induced pluripotent stem cell
MEP: Motor evoked potential
MSC: Mesenchymal stem cell
MRI: Magnetic resonance imaging
NSC: Neural stem cell
OEC: Olfactory ensheathing cell
OPC: Oligodendrocyte progenitor cell
SC: Schwann cell
SCI: Spinal cord injury
SEP: Somatosensory evoked potential
SPSS: Statistical Package for the Social Science
TRH: Thyrotropin-releasing hormone
VAS: Visual analogue scale.
